# Innovative Transcatheter Ventricular Septal Defect Closure Utilizing an Atrial Septal Device in Small Infants: A Case Series

**DOI:** 10.7759/cureus.99720

**Published:** 2025-12-20

**Authors:** Saud A Bahaidarah

**Affiliations:** 1 Pediatric Department, Faculty of Medicine, King Abdulaziz University, Jeddah, SAU

**Keywords:** atrial septal device, case report, small infants, transcatheter closure, ventricular septal defect

## Abstract

Ventricular septal defect (VSD) represents the most prevalent congenital anomaly within the pediatric population. Surgical intervention for closure was the prevailing standard until the late 1980s, when the first transcatheter closure was done. Subsequently, the device industry underwent a transformative evolution, marked by the introduction of a variety of devices aimed at facilitating VSD transcatheter closure. We hereby present two clinical cases involving small infants diagnosed with hemodynamically significant muscular VSD, employing an atrial septal device, accompanied by a detailed procedural description and the resultant outcomes. Presenting two infants were diagnosed with significant congestive heart failure and pulmonary hypertension secondary to large muscular VSD (Case 1, aged 11 months and weighing 5 kg, while Case 2 was 10 months and weighing 5.7 kg). Both patients were subsequently admitted to the catheterization laboratory, where diagnostic cardiac catheterization was performed. Following this, a successful transcatheter closure was achieved via an antegrade approach employing the Amplatzer™ Septal Occluder. In Case 1, six months post procedure, the residual shunt had resolved completely along with the pulmonary hypertension. In Case 2, at a 12-week follow-up, there was a significant decrease in the residual shunt and a reduction in pulmonary hypertension; however, tragically, four months subsequent to the intervention, the patient died due to a COVID-19 infection. The utilization of atrial septal occluders for the transcatheter closure of muscular VSD is achievable during infancy. The selection of an appropriate device size is essential, with the benefits associated with an antegrade approach.

## Introduction

Ventricular septal defect (VSD) constitutes the most prevalent form of congenital heart disease, accounting for approximately 20% of cases [1]. The hemodynamically significant VSDs necessitate surgical closure to avert complications such as left ventricular volume overload, arrhythmias, aortic regurgitation, pulmonary artery hypertension, and the potential for endocarditis [2]. Since the pioneering surgical closure performed by Lillehei et al. in 1954, which established this technique as the gold standard, there have been inherent risks associated with cardiopulmonary bypass, postoperative discomfort, and the resultant sternotomy scar [3,4]. Following the inaugural attempt at transcatheter closure by Lock et al. in 1988 [5], the medical device industry has witnessed significant advancements in varied shapes and materials, accompanied by numerous clinical trials aimed at transcatheter closure of diverse VSD classifications. Initially, muscular VSDs (mVSDs) were addressed with specific mVSD devices; however, numerous other devices, such as Amplatzer Duct Occluder (ADO) I, ADO II, and MFO-Konar, have subsequently been utilized off-label for the closure of such defects, achieving notably high success rates ranging from 83% to 100% [6].

There exist sporadic reports detailing the closure of post-traumatic VSD (PTVSD) in adult patients, as exemplified by the work of Santos et al., who documented a case of a VSD resulting from severe blunt chest trauma sustained in a vehicular accident; this procedure was performed electively after a three-month period, during which the patient remained asymptomatic, despite a significant Qp:Qs ratio, utilizing an antegrade approach with arteriovenous looping [7]. Furthermore, Tang et al. reported a PTVSD that required closure with an atrial septal defect (ASD) device, which regrettably necessitated surgical removal due to uncontrollable hemolysis resulting from a residual defect [8]. In addition, Siagian et al. described a seven-year-old patient presenting with multiple mVSDs and pulmonary hypertension, where an ASD device was employed with the rationale of occluding all VSDs through a single device; a transjugular approach was utilized as outlined by Siagian et al. to mitigate unnecessary curvatures, which was deemed appropriate for the anatomical configuration of the VSDs [9]. Kasem et al. documented a case involving a seven-year-old patient who sustained chest trauma from a truck collision, subsequently complicated by a hemodynamically significant mVSD that was successfully closed using a Cripriform ASD device [10]. To date, there have been no published cases documenting the use of ASD devices for the closure of mVSD in infants.

This report presents two cases in which atrial septal devices were employed for the closure of substantial mVSDs in infants who were underweight for their age.

## Case presentation

Case 1

**Figure 1:**
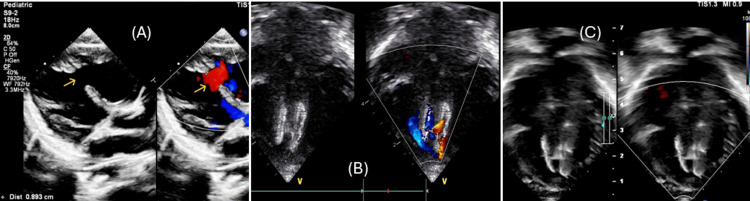
Case 1 echocardiogram images (A) ventricular septal defect (VSD) assessment on echocardiogram on the parasternal long axis view. (B) Device immediately after deployment. (C) Device after six months of deployment on the apical four-chamber view. The yellow arrow shows the VSD.

An 11-month-old female infant was referred to our institution for the assessment of congestive heart failure and a VSD. She presented with clinical manifestations of interrupted feeding, diaphoresis, and significant failure to thrive. Upon physical examination, her weight was recorded as 5 kg (below the third centile for age), alongside a respiratory rate of 40 breaths per minute, tachycardia, the presence of a pansystolic murmur upon auscultation, and hepatomegaly measuring 3 cm below the costal margin (Ross classification class II). Echocardiographic evaluation revealed an 8.5 mm mid-mVSD with indications of pulmonary hypertension, as evidenced by bidirectional shunting across the VSD, as detected by color Doppler imaging (Figure 1A).

**Figure 2:**
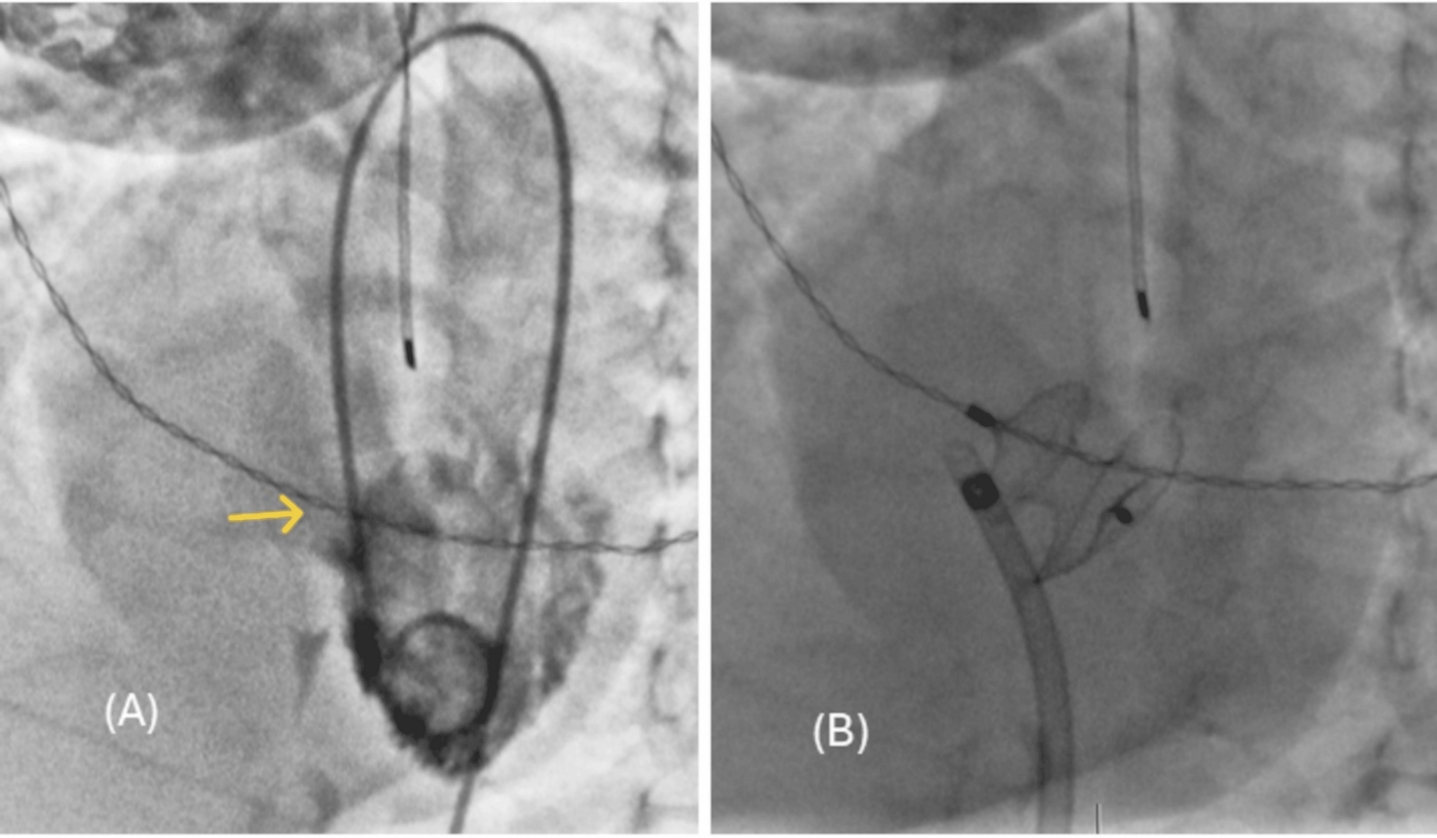
Case 1 catheterization images (A) Ventricular septal defect (VSD) assessment on angiography (four-chamber view). (B) Device on angiography after deployment. The yellow arrow shows the VSD.

The patient was subsequently taken to the catheterization lab, under general anesthesia, where hemodynamic parameters were obtained at FiO_2_ levels of 21% and 100%. The VSD was evaluated through transthoracic echocardiography (TTE) and angiographic imaging (Figure 2A), and it was successfully closed anterogradely using an Amplatzer ™ Septal Occluder size 9 mm delivered via a 6 French Amplatzer™ TorVue™ 180° delivery system (Figure 2B).

Immediate postoperative TTE and follow-up imaging on the second postoperative day demonstrated mild residual flow across the device, along with a decrease in pulmonary artery pressure (TTE showing a left-to-right shunt across the device with a pressure gradient of 35 mmHg) (Figure 1B), and the electrocardiogram indicated a normal sinus rhythm. At the six-month follow-up, the patient remained asymptomatic without any cardiac pharmacotherapy, with an increased weight of 5.8 kg (an increase of 800 g over the six-month period), and echocardiographic assessment revealed appropriate positioning of the device without residual shunting or signs suggestive of pulmonary hypertension at the six-month follow-up (Figure 1C).

Case 2

**Figure 3:**
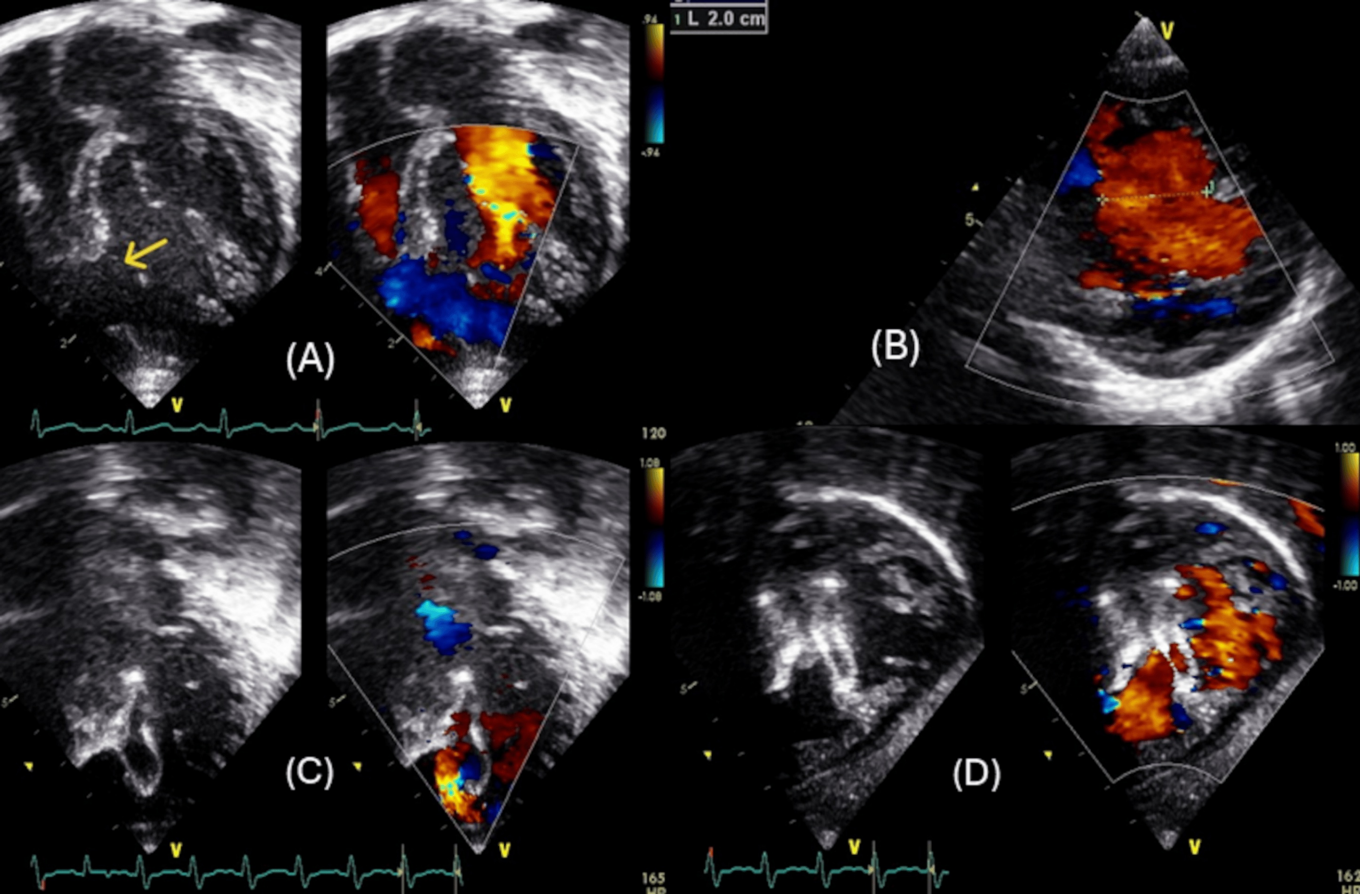
Case 2 echocardiogram images (A)&(B) Ventricular septal defect (VSD) assessment on echocardiogram (apical four-chamber and parasternal short axis views). (C) Device immediately after deployment (apical four-chamber view). (D) Device after 12 weeks of deployment (modified subcostal coronal axis view). The yellow arrow shows the VSD.

A 10-month-old male infant was referred to our institution due to a significant VSD. Upon initial evaluation, he was observed to be in a state of severe heart failure characterized by inadequate weight gain, with a recorded weight of 5.7 kg and recurrent chest infections necessitating hospitalization (Ross classification class IV). Echocardiographic assessment revealed a large muscular VSD measuring at least 12-14 mm, with parasternal short-axis views indicating dimensions reaching up to 20 mm, accompanied by a bidirectional shunt as evidenced by color Doppler imaging (Figures 3A-3B), and a notably dilated left atrium and left ventricle in the context of an ASD.

**Figure 4:**
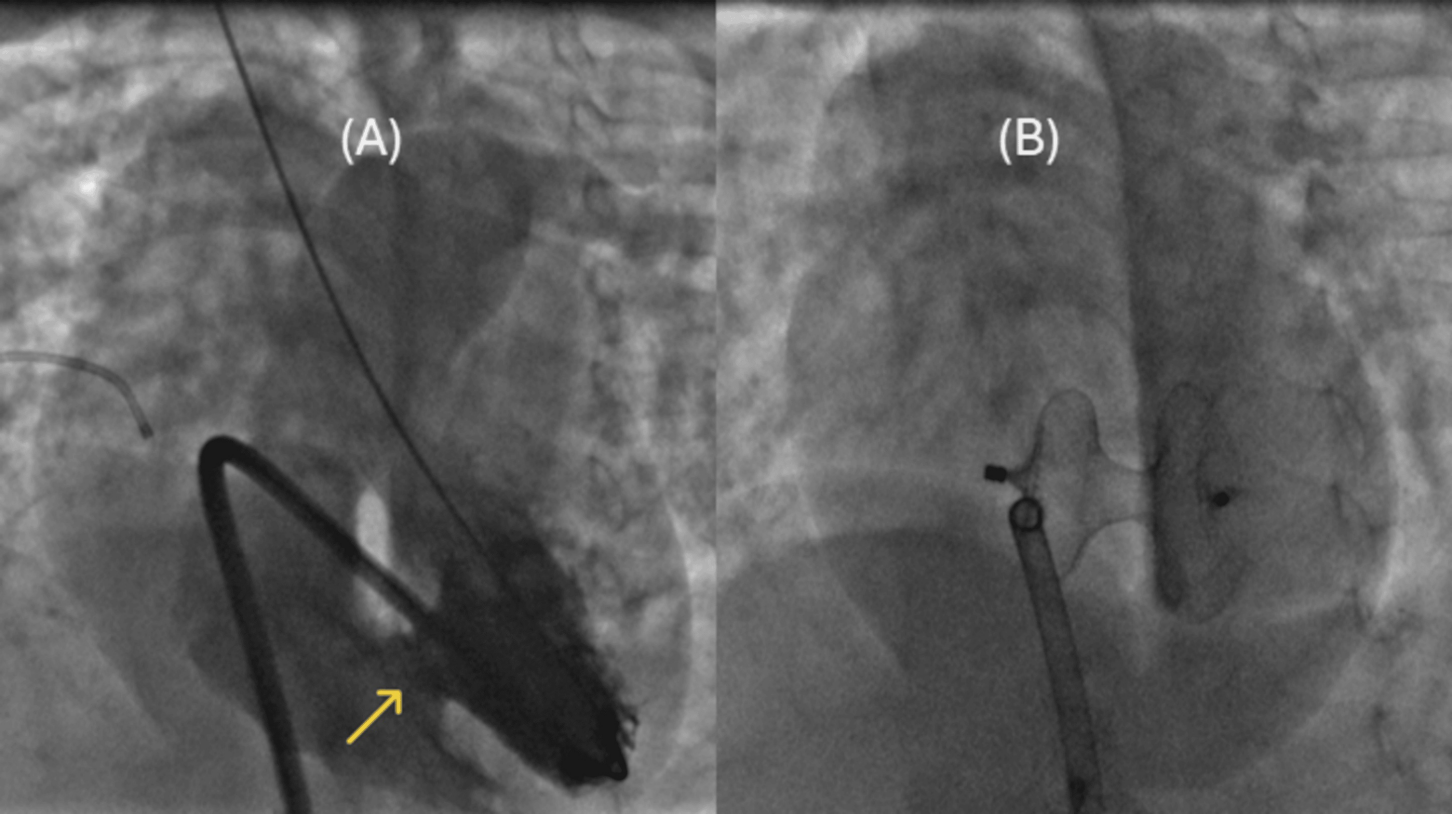
Case 2 Catheterization images (A) Ventricular septal defect (VSD) assessment on angiography (four-chamber view). (B) Device on angiography after deployment. The yellow arrow shows the VSD.

At this juncture, pharmacological interventions and nutritional strategies were optimized to facilitate maximal weight gain in anticipation of transcatheter closure. Following a six-week period, the patient was re-evaluated, demonstrating minimal weight increase (a mere 180 g over six weeks) and continued clinical signs of congestive heart failure. Consequently, the patient was taken to the catheterization laboratory under procedural sedation, where hemodynamic measurements and angiographic studies were conducted. A meticulous assessment utilizing TTE and angiography (Figure 4A) revealed the VSD to measure 18-19 mm at its maximal diameter.

In spite of the presence of pulmonary hypertension, and after careful consideration of the clinical manifestation of heart failure, alongside the dilated left heart structures and small ASD that could potentially function as a pop-off valve in instances of right ventricular hypertension, successful device closure was achieved employing Amplatzer ™ Septal Occluder size 19 mm, delivered via a 9Fr Amplatzer™ TorVue™ 45° delivery system (Figure 4B). Immediate echocardiographic evaluation, along with a follow-up assessment on the subsequent day, indicated moderate residual flow through the device alongside pulmonary hypertension; however, a left-to-right shunt was noted across the ASD (Figure 3C), with the electrocardiogram revealing normal findings, except for the presence of right bundle branch block (RBBB). The patient was readmitted seven weeks subsequent to the catheterization procedure, exhibiting clinical signs of viral pneumonia that necessitated admission to the pediatric intensive care unit (PICU) and the initiation of non-invasive ventilation; follow-up echocardiography revealed systemic level pulmonary artery pressure and a moderate residual shunt through the device, with a total left-to-right shunt across the ASD. Subsequent follow-up at 12 weeks in the outpatient clinic demonstrated a reduction in the residual shunt and pulmonary hypertension, approximating a 50% systemic level of right ventricular hypertension (Figure 3D). Regrettably, four months post procedure, the patient was readmitted to the PICU, requiring mechanical ventilation due to a COVID-19 infection during the early pandemic phase, and subsequently developed multi-organ dysfunction syndrome accompanied by refractory shock, ultimately resulting in demise on the sixth day of hospitalization [11].

Procedure

Patients were admitted one day prior to the procedural intervention and maintained in a nil per os (NPO) state for a minimum duration of six hours preceding the procedure. Within the catheterization laboratory, subsequent to the administration of appropriate anesthesia, access was established to the right femoral artery and vein utilizing 4Fr and 5Fr pediatric femoral sheaths, respectively. Intravenous heparin was administered at a dosage of 100 IU/kg immediately following vascular access, without monitoring for activated clotting time (ACT), as the procedural durations were relatively brief. Right arterial access was employed for the left ventricular (LV) angiogram utilizing a Cordis INFINITI® pigtail 4Fr catheter, as well as for the invasive monitoring of blood pressure throughout the procedure. Biplane imaging was performed using two tubes, with the antero-posterior tube positioned cranially at an angle of 30-35 degrees and the left anterior oblique (LAO) tube similarly angled to adequately profile the interventricular septum (IVS), while the lateral tube was oriented straight. Upon successful completion of the LV angiography, VSD was traversed antegrade utilizing a Cordis INFINITI® JR4 4Fr catheter and a TERUMO GLIDEWIRE® angled .035", 260 cm, which was subsequently exchanged for an EMERALD™ Diagnostic Guidewire .035" J-tip, 260 cm PTFE after catheter positioning in the ascending aorta to facilitate the introduction of the delivery system. Once the delivery system successfully traversed the VSD, it was positioned adjacent to the LV apex, followed by the extraction of the dilator and guidewire (Video 1).

**Video 1:** Angiography through the sheath for ventricular septal assessment

The size of the device was determined to be either equivalent to the size of the VSD or 1 mm smaller than the maximum size measured, a practice informed by previous experiences utilizing ASD devices for VSD closure. Upon loading the device and advancing it to the tip of the delivery sheath, the left disc was revealed near the apex, and the entire system was retracted to align the disc with the IVS following positional confirmation via TTE, at which point the right disc was also uncovered (Video 2).

**Video 2:** Device deployment procedure

The positioning of the device was subsequently verified through TTE and angiography via the delivery sheath with levophase ventriculogram, demonstrating the anatomical contours of both the right ventricular (RV) and LV cavities (Video 3).

**Video 3:** Angiography through the sheath to confirm device position

Any residual shunting through the device was deemed acceptable. The patient was discharged on the second postoperative day following TTE without the necessity of anticoagulants or aspirin therapy.

## Discussion

Although surgical closure of VSD is regarded as the standard therapeutic intervention, families frequently opt for transcatheter closure when feasible, owing to the associated benefits of reduced hospitalization duration, diminished chest pain, and minimized psychological impact of scarring, all while maintaining a comparable success rate [12,13]. Identifying apical and muscular VSDs through a transatrial approach presents significant surgical challenges, often resulting in palliation via pulmonary artery banding (PAB) or ventriculotomy, both of which carry inherent disadvantages [14]. The transcatheter closure of substantial VSDs in small infants poses notable difficulties due to the potential risks of hemodynamic instability and arrhythmias [15]. Various devices have been developed for the management of these defects, such as the Amplatzer mVSD device and Konar MFO, along with off-label applications of ADOI and ADOII [12,13,15]. The Amplatzer device for ASD is characterized by its low profile, consisting of dual discs made from nitinol mesh and polyester material, featuring a waist of 4 mm, in contrast to the VSD device, which is constructed with a 7 mm waist to accommodate the IVS with symmetrical discs, while the ASD device incorporates a smaller right disc compared to the left. During the deployment of the ASD device, it was observed that it easily conformed to the thick IVS, with the low-profile right disc positioned within the trabeculated RV, even in the presence of a moderator band, which represents a surgical challenge [16].

In contrast to the documented instances of muscular VSDs being occluded using ASD devices, where retrograde and A-V looping techniques or transjugular approaches were employed [7-10], an antegrade approach was utilized to traverse the VSD to position the delivery sheath at the LV apex, thereby avoiding injury to the aortic valve and preventing the formation of an arteriovenous loop that prolongs fluoroscopy and procedural duration. Additionally, the stretching of the aortic and tricuspid valves with a loop in smaller hearts could potentially lead to hemodynamic instability [17].

TTE was employed for the assessment and closure of VSDs in conjunction with fluoroscopy in both cases, owing to the clarity of imaging achievable in this young patient demographic, as documented in the literature alongside TEE [18]. The parasternal short-axis view is recognized as an effective perspective for measuring the maximum defect size. Based on our experience utilizing the ASD occluder for transcatheter VSD closure, it is recommended that the VSD size be equivalent to or 1 mm smaller than the maximum measurement obtained through any modality, thereby facilitating a reduced stretching of the device.

The limitation of these reports, besides being retrospective, was that the long-term outcome was not assessed, as one of the cases expired, and the second case was followed for only a short time.

## Conclusions

The utilization of ASD occluders for the transcatheter closure of mVSD is achievable during infancy, particularly in instances of large defects that may pose significant surgical difficulties for complete closure. The selection of an appropriate device size is essential, with the benefits associated with an antegrade approach being notable. A rise in the volume of reports and studies is necessary to fully explore the efficacy and safety of these devices in off-label contexts.
